# Construct validity of the physical activity neighborhood environment scale-Oman

**DOI:** 10.3389/fpubh.2023.1007075

**Published:** 2023-03-17

**Authors:** Gustavo De Siqueira, Ruth Mabry, Huda Al Siyabi, Ahmad Adeel, Sadmira Malaj, Adewale Oyeyemi

**Affiliations:** ^1^Department of Urban Planning and Architectural Design, German University of Technology, Muscat, Oman; ^2^Independent Public Health Researcher, Muscat, Oman; ^3^Department of Community-Based Initiatives, Ministry of Health, Muscat, Oman; ^4^College of Health Solutions, Arizona State University, Phoenix, AZ, United States; ^5^Department of Physiotherapy, Redeemer's University, Ede, Nigeria; ^6^Department of Physiotherapy, University of Maiduguri, Maiduguri, Nigeria

**Keywords:** physical activity, active mobility (walking and cycling), public and open spaces, construct validity, healthy cities, Oman, digital methodologies

## Abstract

**Aims:**

This study aims to examine the construct validity of Physical Activity Neighborhoods Environment Scales, Oman (PANES-O), and compare the subjective perceptions with objective measures in Muscat, the capital area of Oman.

**Methods:**

Walkability index scores using GIS maps were calculated for 35 study areas in Muscat based on which five low and 5 high walkable study areas were randomly selected. A community survey was then conducted in November 2020 in each study area using the 16-item PANES-O instrument to measure the participants' perception of neighborhood density, land use mix, infrastructure, safety, aesthetics, and street connectivity. Due to pandemic restrictions, a social media-based purposive sampling strategy was utilized to reach community-based networks and complete digital data collection.

**Results:**

Significant differences between the low and high walkablehigh-walkable neighborhoods were observed for 2 of 3 macroenvironment subscales, density and land use. Respondents in high walkable neighborhoods perceived their areas as having more twin villas (*P* = 0.001) and apartment buildings (*P* < 0.001), greater access to destinations (like more shops, and places to go within walking distance; *P* < 0.001), easy access to public transport (*P* < 0.001), and more places to be active (*P* < 0.001); than their counterparts in low walkable neighborhoods. In terms of microenvironmental attributes, respondents in high walkablehigh-walkable neighborhoods perceived their areas to have better infrastructure, better aesthetic qualities, and better social environment than their counterparts in low walkablelow-walkable neighborhoods. Significant differences in perceptions across 12 of the 16-item PANES tool confirmed that 6 of the 7 subscales were significantly sensitive to built environment attributes between the low and high walkable study areas. Respondents in high walkable neighborhoods perceived their areas as having greater access to destinations (like more shops, places to go within walking distance; *P* ≤ 0.001), easy access to public transport (*P* ≤ 0.001), more places to be active (*P* ≤ 0.001), better infrastructure (like more sidewalks, facilities to bicycle; *P* ≤ 0.001), and better aesthetic qualities (*P* ≤ 0.001). PANES-O also was able to rate high walkable neighborhoods to be higher in residential density and land-use mix compared to the low walkable neighborhoods demonstrating its sensitivity to the GIS maps' objective measures.

**Conclusions and recommendations:**

These results provide preliminary strong support for the construct validity of PANES-O, suggestingconfirming that it is a promising tool for assessing macroenvironmental perceptions related to physical activity in Oman. Further research using objective measures of microenvironments and device-based physical activity scores is needed to confirm the criterion validity of the 10 micro-environmental attributes of PANES-O using objective measures for the microenvironment. PANES-O could be used to generate and develop the needed evidence on the most appropriate approaches to improving the built environment to promote physical activity and urban planning in Omanthe country.

## 1. Introduction

Urbanization and modernization have changed the urban landscape in Oman; residential neighborhoods have ben designed for cars rather than active transport like walking and cycling (1–3). Sedentary lifestyle and associated physical inactivity-related non-communicable diseases are now major public health burden in Oman (4, 5). Physical inactivity is a key modifiable behavioral risk factor for multiple non-communicable diseases (6). Built environment interventions to increase residential, intersection and transit density, land-use mix, and public transport transit and park access are effective strategies to improve physical activity and reduce sedentary time (7, 8). Such interventions can provide long-term impact on physical activity on a permanent basis and affect virtually the entire population (9, 10). However, little is known about how built environment interventions can be used to guide urban planners and public health decision-makers to create an environment that supports active living in Oman.

Some efforts have been made to improve neighborhood walkability in Oman. The Where Oman Walks (WOW) project was initiated by a research team atof the German University of Technology in Oman in 2018 (11). It aims to develop urban regeneration strategies with a focus on pedestrianization of the residential neighborhoods in the Muscat capital area. The WOW team works in collaboration with partners of different sectors including the World Health Organization, Oman, the Ministry of Health, Oman, and the Muscat Municipality to pilot some interventions including a small-scale urban regeneration project in the neighborhood of the Ammar Bin Yaser Mosque in Muscat. Yet, more evidence on micro-scale environmental attributes isare needed to inform effective urban and public health planning in Oman and the Middle-EastMiddle East and African (MENA) region.

The international physical activity prevalence study (IPS) group developed a 17-item tool, Physical Activity Neighborhoods Environment Scale (PANES), to assess the micro-scale environmental factors relevant for to walking and bicycling in communities (12). It assesses perceptions relevant to walking and bicycling such as land use mix, residential density, pedestrian infrastructure, aesthetic qualities, and safety from traffic and crime (13). This tool has been used to assess the built environment and physical activity in a range of countries globally but not in the Arab world (12, 14). An earlier study described the adaptation of PANES to the Omani context (PANES-O) and assessed its test-retest reliability (15). The test-retest reliability of the PANES-O was acceptable with Intraclass Correlation Coefficients (ICCs) ranging from 0.436 to 1.000 for all items. Yet, no validity evidence has been reported for PANES-O. Because accurate analyses of the built environment and physical activity relationships require the use of valid measures of attributes of the built environment as well as of physical activity (16), the present study aims to examine the construct validity of PANES-O and compare the subjective perceptions with objective measures in Muscat, the capital area of Oman.

## 2. Methods

Construct validity aims to test whether the PANES-O tool is sensitive enough to capture the environmental differences across existing neighborhoods in Muscat. Thus, this study follows a three step process: (1) selecting study areas from different districts in Muscat Governorate, (2) identifying low and high walkable study areas based on objective mesurements, and (3) conducting a community survey to compare the subjective measures between high and low walkability areas.

### 2.1. Selecting and defining study areas

**Figure 1:**
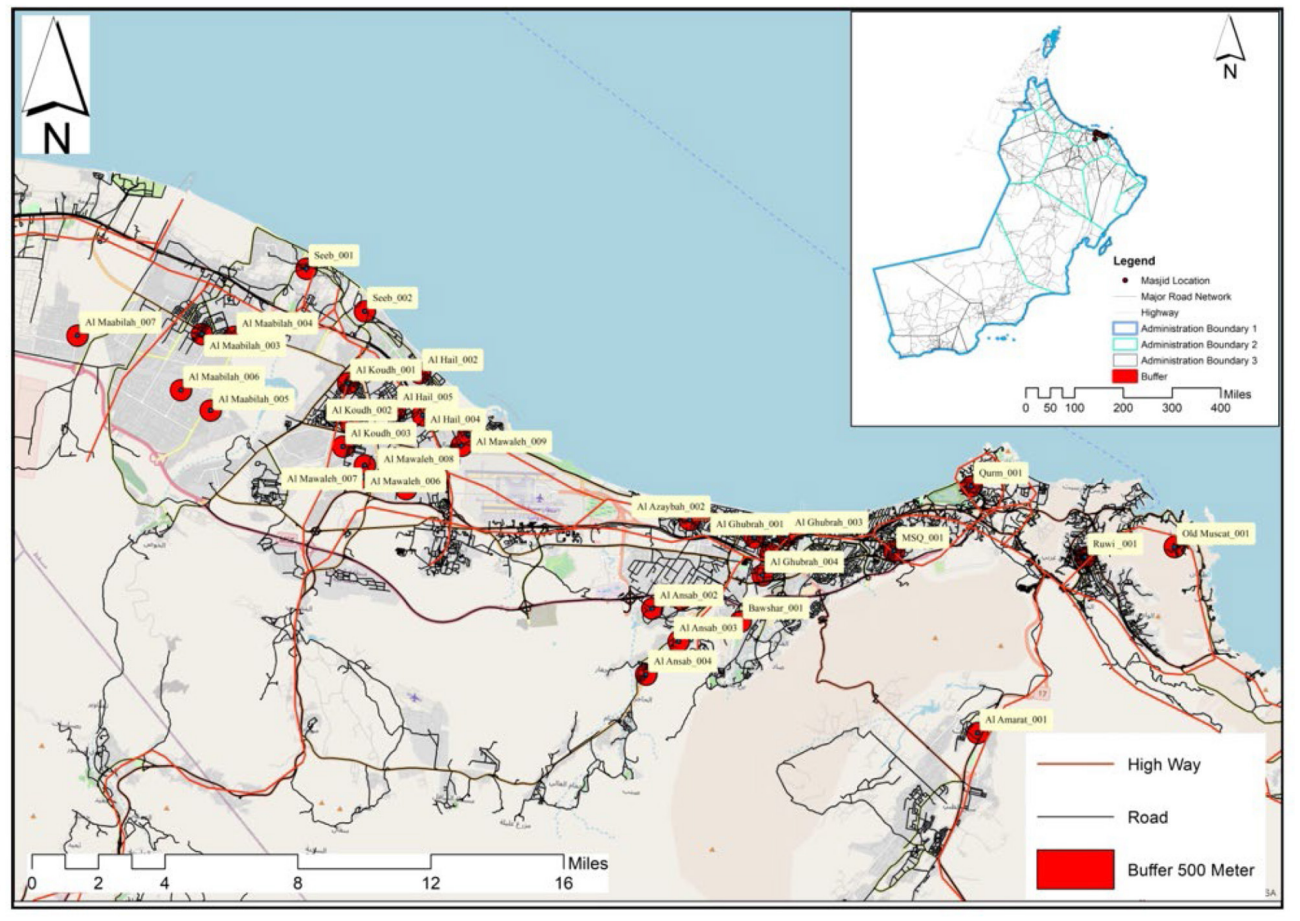
Initial selection of potential study areas, Muscat, Oman.

To capture a diverse range of environmental attributes, 35 study areas were selected from 5 districts of Muscat using three selection criteria. (1) Residential areas with at least 50% of the plots occupied (Figure 1). High demands due to the land allocation system to provide each Omani of 23 years of age and above with a plot of land has led to scattered development with some areas remaining vacant or sparsely developed. (2) Areas with large voids (e.g., wadis and dry riverbeds), crossed by highways, and those covered by previous versions of the WOW project were excluded. (3) Areas with a straight-line buffer of 500 m (5-min walk) around the local mosque. In Oman, neighborhood mosques are evenly distributed, usually a walkable distance for area residents (11) and work as a community gathering point.

### 2.2. Defining low and high walkable study areas

The An objective assessments was used to develop the walkability score for the study areas. This involved included drafting GIS maps from each selected neighborhood addressing the three macro-level environment indicators (residential density, street connectivity, and land use mix). The three micro-level environment indicators related to the streetscape (pedestrian infrastructure, safety, and aesthetics) (17, 18) are not considered in this step. Maps were designed based on satellite images and completed with measurements on-site due to limited access to a central repository.

For each of the 35 study areas, we calculated the Walkability Index Score (18) by adding the standardized values (*Z*-scores) of dwelling units' density (as representative for residential density), land use mix entropy and the density of street intersections with more than 2 junctions (as a measurement for street connectivity) without weightings. Based on this score, we divided them into 6 higher (WS ≥ 0.9) and 29 lower walkability study areas (WS < 0.9). We then randomly selected five low walkable and five high walkable study areas (Table 1) to equalize the size of two independent samples to avoid loss of statistical power (19).

**Table 1:** Walkability scores of the selected study areas.

**District (neighborhood)**	**Land use mix**	**Street connectivity (intersect density)**	**Residential density**	**Walkability index**
Al Hail _005^*^	0.12	0.89	2.32	3.33
Qurm_001^*^	1.44	−1.03	1.85	2.26
Seeb_001^*^	1.77	0.23	0.14	2.14
Al Ghubrah_004^*^	1.29	0.23	0.52	2.04
Al Ghubrah_001^*^	−0.68	0.96	0.68	0.97
Al Hail _004^**^	−0.55	0.30	0.09	−0.16
Al Mawaleh_009^**^	0.50	−0.07	−0.86	−0.42
Al Mawaleh_006^**^	−0.43	0.30	−0.43	−0.56
Al Maabilah_005^**^	−0.98	0.60	−0.78	−1.16
Al Mawaleh_004^**^	−1.40	−0.58	−0.91	−2.90

* Higher.

** Lower walkability scores.

### 2.3. Conducting community survey

#### 2.3.1. Sampling and recruitment

We carried out a community survey of participants in the 10 selected neighborhoods. A purposive sampling strategy was used to recruit a broad group of women and men from different age groups, the two key demographic characteristics influencing physical activity in Oman (5). Data collection was conducted in November 2020. Due to restrictions imposed by the Supreme Council during the COVID-19 pandemic that discouraged in-person household surveys, the research team created a digital survey in Survey Monkey (20) with links (one version in Arabic and one in English) for each study area.

**Figure 2:**
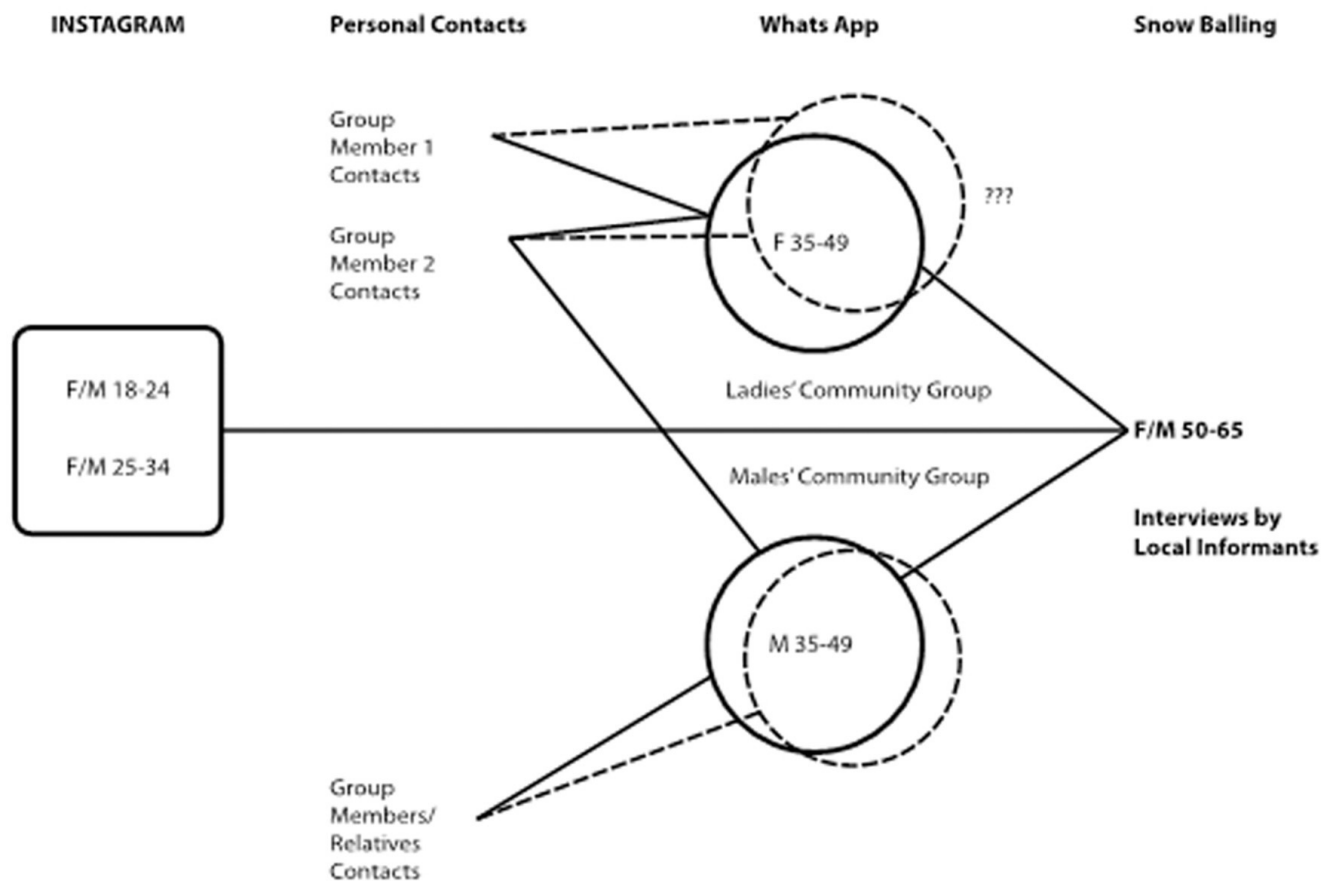
Schematic representation of the survey dissemination strategy by target groups (age and sex) and dissemination means.

The ubiquitous use of mobile phones and social media provided alternative approaches to in-person interviews during the social interaction restrictions brought on by the pandemic. To address potential bias—as digital devices are more commonly used by the younger population—three sampling strategies were used to reach community-based networks, especially those restricted to the geographic boundaries of the study areas instead of, for example, family groups that are likely to be spread in different locations (see also Figure 2).

- To target young adults (<29 years of age), a message was posted in three different Instagram accounts (including the “Where Oman Walks” Instagram project account) inviting volunteers living in one of the study areas to contact the team specifying which area they reside. The volunteers would then receive a specific link to the on-line survey for the specific study area.
- To target women and men (30–49 years), Whatsapp messages about the survey were sent to a variety of geographically restricted and gender-segregated groups including neighborhood mosque groups (usually composed of senior and middle-aged men), Quran school groups (usually include women of all ages), and sports groups (young women and men).
- To target women and men over 50 years of age who are less likely to be reached by Instagram and Whatsapp groups, a snowballing method was employed. Younger respondents living in multigeneration households were invited to conduct an interview and complete an on-line survey for an older member of the household.

#### 2.3.2. Data collection methods

Data were collected in the Fall of 2020 by a group of 98 trained research assistants who are students of the Urban Design 2 course in the German University of Technology, Oman. The research assistant received a 2 weeks training that entailed descriptions of the purposes of the study, training on data collection techniques, ethical issues, and how to interpret the results. They were supervised by GdS and SM within the framework of the educational branch of the WOW project (De Siqueira and Al Balushi 2020). The majority of the data collectors were young (20.5 years old) and female (*N* = 97).

#### 2.3.3. Ethics and methodological considerations of digital data collection

The use of social network tools for research purposes has the advantage of providing fast and inexpensive access to a wealth of data. However, it also poses several challenges. Social media depends on mobile devices and internet access which might exclude population sub-groups due to economic restrictions. Additionally, in some cultural contexts access to social media is a privilege for male users (21). Studies using social media raise concerns about data privacy and informed consent (22, 23). Thus, for this study, WhatsApp was deployed as a means to disseminate the access link to an electronic survey in SurveyMonkey; formal consent was obtained once participants accessed the survey link.

#### 2.3.4. Information collected

To assess participants perceptions of the built environment we used the 16-item PANES-O tool; its adaption and testing was described in our earlier study (15). In summary, the adaptation involved revision by local experts, Arabic translation, and cognitive testing. The reliability testing with a sample of Omani women and men demonstrated good level of consistency. This tool assesses perception of neighborhood density, land use mix, infrastructure, safety, aesthetics and street connectivity. In addition, information regarding transport physical activity and demographics including age (18–29, 30–39, 40–49, and 50–65 years), sex, marital status (single, married, separated, divorced, and widowed), and educational level (can read and write, primary school, preparatory school, general diploma, high school diploma, and university degree or higher) was obtained from the participants.

For transport physical activity we include three items from the International Physical Activity Questionnaire; namely. a dichotomous question to define active transport users (During the last 7 days... Do you Walk or cycle for at least 10 min?), the average frequency of active transport days (In a typical week, on how many days do you walk or bicycle for at least 10 min continuously to get to and from places?), and the average daily activity duration (How much time do you spend walking or bicycling for travel on a typical day?) (24).

### 2.4. Analysis

Bivariate analysis was conducted to compare perceptions between the low and high walkable neighborhood groups. Demographic and perceived neighborhood environment differences between residents in the two groups were examined using independent *t*-tests for continuous variables and Chi-square for dichotomous variables. For the test of the construct validity, which investigates the tool's ability to distinguish between neighborhoods with dissimilar features, analysis of covariance tests adjusted for sex and age were conducted; it examines neighborhood differences in perceived environment attributes (14). Statistical analysis was performed employing SPSS v. 17 at an alpha = 0.05 confidence level.

## 3. Results

A large portion of the sample (61%) were young (age group 18–29 years) and female (61%). More than half were single (55%), and a large portion were university degree holders (49%) (see Table 2). These figures are fairly similar to the population distribution in Oman in terms of sex (57% female) and age (25.2% age 40–49 and 50–65 years combined) (25). Underrepresentation of male respondents was higher in low walkable neighborhoods (36.9%) than in the high walkable neighborhoods (42.9%). The concentration of responses around the 18–29 age group was higher in the high walkable neighborhoods (65.1/58.2%). Respondents in high walkable study areas reported slightly higher levels of transport physical activity across the three physical activity related questions.

**Table 2:** Sample demographics.

**Description**	**%**		
	**Low walkable neighborhood (*****n*** = **423)**	**High walkable neighborhood (*****n*** = **287)**	**Total sample (*****N*** = **713)**
**Sex**			
Male	36.9	42.9	39.3
**Age (years)**			
18–29	58.2	65.1	61.0
30–39	13.3	14.5	13.8
40–49	18.1	14.5	16.6
50–65	10.5	5.9	8.6
**Civil status**			
Never married	51.8	60.2	55.2
Married	44.0	33.2	39.6
Separated/divorced/widowed	1.9	2.4	2.1
Other	2.4	4.2	3.1
**Educational level**			
General diploma or less	21.5	22.4	21.9
Highschool diploma	29.6	27.3	28.7
University degree or higher	48.9	50.2	49.4
**Transport physical activity**			
Walk/Cycle at least 10 min continuously last week	37.7	46.0	42.6
Average number of days walk/cycle at least 10 min continuously (days)	2.7	2.8	2.7
Average time spent walking/cycling for transport/week (mins)	26.1	30.1	28.4

For construct validity, significant differences between the low and high walkable neighborhoods were observed for density and land use, two of the three macroenvironmental attributes measured by the Walk Score 12 of the 16 items of PANES-O including the 4 sub-items of neighborhood density (Table 3). Respondents in high walkable neighborhoods perceived their areas as having more twin villas (*P* = 0.001) and apartment buildings (*P* < 0.001), greater access to destinations (like more shops, places to go within walking distance; *P* < 0.001), easy access to public transport (*P* < 0.001), and more places to be active; *P* < 0.001), than their counterparts in low walkable neighborhoods. Respondents from low walkable neighborhoods perceived their neighborhoods to have more single family villas (*P* = 0.05) and town/row houses (*P* = 0.041) than those from high walkable neighborhoods. There were no significant differences in the mean scores for items related to street connectivity.

**Table 3:** Difference in mean (SD) scores of the physical activity neighborhood environment scale—Oman by low and high walkability neighborhoods in Muscat, Oman.

**Item**	**Items**	**Mean (SD)** ^**a**^		***F*-value**	**Adj. *R*^2b^**	***P*-value**
		**Low walkability neighborhoods**	**High walkability neighborhoods**			
**Density**						
1	1.1 Single family villas	3.00 (0.55)	2.96 (0.63)	2.611	0.007	0.050
	1.2 Twin villas	2.41 (0.67)	2.61 (0.66)	5.837	0.020	0.001
	1.3 Town/row houses	1.94 (0.70)	1.83 (0.70)	2.766	0.037	0.041
	1.4 Apartment buildings	2.15 (0.74)	2.32 (0.80)	6.478	0.023	< 0.001
**Land use and access to destinations**						
2	Many shops, stores, markets or other places to buy things I need are within easy walking distance of my home.	1.86 (0.9)	1.94 (0.90)	14.602	0.055	≤ 0.001
3	There are many places to go within easy walking distance of my home such as mosques, schools, health institutions, workplaces, markets, parks, etc.	1.84 (0.85)	1.86 (0.83)	7.361	0.027	≤ 0.001
4	It is within easy walking distance from my home to access public transport and taxi on the main road of my neighborhood.	2.29 (0.97)	2.61 (1.01)	12.889	0.048	≤ 0.001
5	My neighborhood has several places such as open fields, parks, sea, clubs, and gymnasium to exercise and play football and other sports.	2.28 (1.01)	2.84 (1.03)	20.662	0.078	≤ 0.001
**Pedestrian infrastructure**						
6	There are sidewalks on most of the streets in my neighborhood.	2.15 (1.06)	2.56 (1.11)	19.639	0.075	≤ 0.001
7	There are facilities to bicycle in or near my neighborhood, such as special lanes, separate paths, shared-use paths for cycles, and pedestrians.	2.93 (1.02)	3.12 (1.00)	6.730	0.024	≤ 0.001
8	Places for bicycling (such as bike paths) in and around my neighborhood are well maintained and not obstructed	3.14 (0.92)	3.25 (0.94)	5.002	0.017	0.002
9	The sidewalks in my neighborhood are well maintained (paved, with few cracks) and not obstructed	2.47 (0.93)	2.79 (1.00.)	11.397	0.043	≤ 0.001
**Neighborhood safety**						
10	Walking during the day is safe in my neighborhood.	1.61 (0.79)	1.70 (0.85)	13.051	0.001	0.050
11	Walking at night is unsafe in my neighborhood.	2.70 (0.98)	2.75 (1.01)	1.489	0.002	0.216
12	There is so much traffic on the streets that makes it difficult or unpleasant to walk in my neighborhoods.	2.43 (1.01)	2.41 (1.02)	1.110	0.022	0.344
13	There is so much traffic on the streets that it makes it difficult or unpleasant to ride a bicycle in my neighborhood.	2.22 (0.96)	2.18 (0.99)	2.306	0.006	0.076
**Neighborhood aesthetics**						
14	There are many interesting things to look at while walking in my neighborhoods such as shady trees, building variety, beautiful beaches, etc.	2.37 (1.03)	2.73 (1.00)	8.580	0.032	≤ 0.001
**Street connectivity**						
15	There are many cross-junctions in my neighborhood.	1.91 (0.77)	1.86 (0.80)	1.046	0.002	0.371
**Social environment**						
16	I see many people being physically active in my neighborhood doing things like walking, jogging, cycling, or playing sports and active games	1.68 (0.76)	1.73 (0.76)	2.932	0.061	0.033

a Mean (SD) scores based on a 4-point Likert scale. Items 11–13 are reversed;

b R^2^ adjusted for sex and age.

In terms of microenvironmental attributes, respondents in high walkable neighborhoods perceived their areas to have better infrastructure (like more sidewalks, facilities to bicycle; *P* < 0.001), safer to walk during the day (*P* = 0.05), better aesthetic qualities(*P* < 0.001), and better social environment (“many people being physically active”; *P* = 0.033) than their counterparts in low walkable neighborhoods. However, there were no significant differences in the mean scores for and almost all items on neighborhood safety (except walking during the day). Respondents in high walkable neighborhoods perceived better safety for walking during the day than their counterparts in low-walkable neighborhoods.

## 4. Discussion

This study builds on our earlier article describing the adaptation of the Physical Activity Neighborhood Environment Scale in Oman (PANES-O) that demonstrated good test-retest reliability. The main finding of the present study is that 2 of 3 macroenvironment subscales 6 of 7 subscales and 12 of 165 of 6 items of the PANES-O were significantly sensitive to the objective measures of built the macroenvironment attributes used in the Walk Scorebetween low and high walkable neighborhoods. These results provide strong support for the construct validity of PANES-O, suggestingconfirming that it is a promising tool for assessing environmental perceptions related to physical activity in Oman.

The traditional components of walkability are residential density, street connectivity and land-use mix (17, 18). Consistent with the traditional definition of walkability and findings in Western countries (26, 27), the PANES-O was able to rate perceptions of participants living in high walkable neighborhoods in Oman to be higher in residential density and land-use mix than those of residents in low walkable neighborhoods. Street connectivity was the only major characteristics of neighborhood walkability found not to be rated differently by participants from low and high walkable neighborhoods. Similar discrepant finding for street connectivity has been reported for the PANES and other built environment measures in multiple African countries (14, 16). Perhaps, the concept of street connectivity has a different meaning and could be interpreted differently across culture and continents.

Worthy of note is that PANES-O was sensitive to other activity friendly attributes of the microenvironments beyond the traditional components of walkability. Several favorable built environment indicators that were rated higher by residents of high walkable neighborhoods includes ease of access to public transport, more places to be active, better infrastructure (like more sidewalks and places to bicycle), safety to walk during the day, better aesthetic qualities, and better physical active social environment. This ability of the PANES-O to discriminate differences in built microenvironment attributes across the selected low and high walkable neighborhoods provides strong support for its construct validity and utilityrequires further confirmation by testing specific associations of the microenvironment attributes with objectively assessed neighborhood walkability and socio-economic status. A similar approach has been used to confirm the construct validity of the neighborhood environment walkability scale (NEWS) measureusing an appropriate tool for objective measures such as the neighborhood environment walkability scale (28).

The most significant differences in perceptions of built environment attributes were related to three attributes; land use mix, pedestrian infrastructure and social environment confirming the importance of the WOW project in promoting the pedestrianization of residential neighborhoods (11). The importance of neighborhood infrastructure supportive to being physically active, particularly the need for public spaces away from vehicular traffic was emphasized by participants in earlier qualitative studies carried out in the country (5, 29). Oman is ranked among the safest countries globally for tourists (30) and reported violent crime is similar to neighboring countries in the Arabian Gulf (Bahrain: 1.0, Oman: 0.9 and Qatar: 0.3 per 100,000 population) and markedly lower than other countries in the region including Morocco, Jordan and Egypt (1.3, 2.1 and 4.5 per 100,000 population, respectively) (31) thus, it is not surprising that perceptions of safety did not vary across neighborhoods.

Findings of good validity and reliability evidence for PANES-O have broad implications for urban planning and physical activity research in Oman. PANES-O would be useful for developing and strengthening the evidence on the most cost-effective and socio-culturally appropriate approaches to improving the built environment to promote physical activity in the country. This is an urgent urban and public health priority for Oman as the current national physical activity plan of action for the country is weak on built environmental interventions for active living (32). Although respondents from high walkable study areas reported doing more transport related physical activity compared to those living in low walkable areas, further research of the association of both transport and leisure physical activity and the built environment in Oman is needed to better guide public health action (12).

This study has some important limitations that must be acknowledged. The use of digital technologies and social media and having a young research team, predominantly young (18–29 years) female undergraduate students, may have been some of the reasons the sample was also young and highly educated. On the other hand, the purposive and large sample size ensured a good gender balance which addresses concerns of male bias in use of digital technologies (21); the recruitment strategy also successfully recruited people in older age groups that are similar to the general population in Oman. Although the study setting was just in the capital area of Oman, selecting samples from low and high walkable neighborhoods using objective measures of walkability ensured sufficient variation in the built macro-environment. However, further research is needed to confirm the validity of the 10 micro-environmental attributes of PANES-O. The Walk score does not capture microenvironments, so it is important for future studies to use objective microenvironment measures and device-based physical activity scores to quantify the criterion validity of PANES-O using objective measures for the microenvironment such as the neighborhood environment walkability scale (28, 33).

In conclusion, the adapted PANES-O demonstrated good some evidence of construct validity and is sensitive to objective measures of macroenvironmental variations. It is a promising tool for assessing environmental perceptions related to physical activity in Oman and possibly other countries with similar built environments and culture in the Middle East and Africa (MENA) region.

## Data availability statement

Data supporting the conclusions of this article will be made available on request.

## Ethics statement

The studies involving human participants were reviewed and approved by German University of Technology in Oman. The patients/participants provided their written informed consent to participate in this study.

## Author contributions

All authors listed have made a substantial, direct, and intellectual contribution to the work and approved it for publication.
